# Galactose‐Functionalized Gold Nanoparticles Targeting Membrane Transporters for the Glutathione Delivery to Brain Cancer Cells

**DOI:** 10.1002/cbic.202500104

**Published:** 2025-06-17

**Authors:** Francesca Milano, Alessia Nito, Annalisa Caputo, Antonio Gaballo, Marco Marradi, Alessandra Quarta, Andrea Ragusa

**Affiliations:** ^1^ Department of Chemistry ‘Ugo Schiff’ University of Florence via della Lastruccia 3‐13 50019 Sesto Fiorentino (FI) Italy; ^2^ CNR NANOTEC Institute of Nanotechnology Campus Ecotekne 73100 Lecce Italy; ^3^ Department of Engineering for Innovation University of Salento Campus Ecotekne 73100 Lecce Italy; ^4^ Department of Mathematics and Physics University of Salento Campus Ecotekne 73100 Lecce Italy; ^5^ Department of Life Sciences Health and Health Professions Link Campus University Via del Casale di San Pio V 4400165 Rome Italy

**Keywords:** GLUT, glutathione, gold nanoparticles, oxidative stress

## Abstract

Glutathione (GSH), a tripeptide essential for maintaining redox balance in the human body, plays a critical role in protecting cells from oxidative stress. A deficiency in GSH is linked to increased oxidative damage and the progression of various disorders, including cancer and neurological diseases. Herein, gold nanoparticles (Au NPs) coated with GSH and further functionalized with galactose moieties are developed to selectively target glucose transporters (GLUT), which is overexpressed on the surface of the blood‐brain barrier (BBB) and could be exploited for the selective recognition and internalization of the Au@GSH‐Gal NPs, that could then exert an antioxidant effect. As a proof of concept, brain cancer cells are treated with Au@GSH‐Gal NPs, evidencing their increased internalization and a significant reduction of H_2_O_2_‐induced oxidative stress.

## Graphical abstract

Glutathione‐functionalized gold nanoparticles are shown to reduce radicals in SH‐SY5Y and C6 cells. Conjugation of galactose to the outer shell improves internalization through the GLUT transporter, thus providing a smart multivalent system able to reduce oxidative stress in neuroblastoma and rat glioma cells.

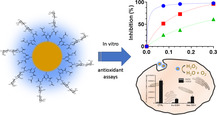

## 1 Introduction

The treatment of brain disorders, including brain cancers and neurodegenerative diseases such as Alzheimer's, Parkinson's, and Huntington's, remains one of the most compelling challenges in modern medicine. Existing therapies are often only partially effective and frequently accompanied by significant side effects of current anticancer drugs.^[^ ^1^ ^]^ A major problem in the therapeutic treatment of brain diseases is the fact that drugs must be able to cross the blood–brain barrier (BBB), which represents an insurmountable wall for many molecules.^[^ ^2^ ^]^

Reactive oxygen species (ROS) are naturally occurring molecules generated as a byproduct of normal cellular metabolism. However, when present in excess, they can induce damage to cells, tissues, and organs. Oxidative stress, a condition in which ROS production in the body exceeds the ability of the antioxidant defense systems to neutralize them, has been implicated in a wide range of diseases, including cancer, neurodegenerative disorders, cardiovascular diseases, and diabetes.^[^ ^3^ ^]^ A growing body of evidence has also revealed the role of ROS in promoting drug resistance in brain tumors.^[^ ^4^ ^]^ The body employs several defense mechanisms to counteract the detrimental effects of oxidative stress, among which glutathione (GSH), a tripeptide molecule that acts as a powerful antioxidant.^[^ ^5^ ^]^ Beyond its direct radical scavenging properties, GSH also facilitates the regeneration of other crucial antioxidants, such as vitamins C and E, thereby enhancing the overall antioxidant capacity of cells. Notably, in patients with Parkinson's disease, a reduced concentration of GSH has been observed in the substantia nigra, correlating with an increase in oxidative stress, elevated ROS generation, and subsequent loss of dopaminergic neurons.^[5a]^ GSH activity has also been linked to brain cancer progression and chemoresistance, wherein GSH and its related enzymes, such as glutathione peroxidase (GPx) and glutathione *S*‐transferase (GST), are frequently dysregulated.^[^ ^6^ ^]^ In this regard, GSH supplementation could help to reduce oxidative stress and modulate cancer activity.^[^ ^7^ ^]^ Nevertheless, exogenous supplementation of antioxidant molecules has to face pharmacokinetic issues, having difficulties in reaching the desired target and being easily degraded and excreted before exerting their therapeutic role. Similarly, systemic delivery of GSH is hindered by poor stability and limited transport across the BBB.

The use of nanomaterials to improve the diagnosis, treatment, and prevention of diseases has already emerged as a promising approach for the development of innovative therapeutics.^[^ ^8^ ^]^ Indeed, nanoparticles (NPs) can be engineered to possess specific attributes, such as size, shape, and surface chemistry, which enable controlled and precise interactions with biological systems. Furthermore, NPs can increase therapeutic effectiveness and reduce side effects by selectively delivering drugs to diseased cells.^[^ ^9^ ^]^ NPs have also been successfully exploited to alleviate oxidative stress, overcoming the well‐known pharmacokinetic limitations of antioxidant molecules.^[^ ^10^ ^]^ The use of GSH delivery systems, such as liposomes, NPs, and dendrimers, has also demonstrated the protection of the small peptide against inactivation mechanisms and systemic clearance.^[^ ^11^ ^]^ Among them, gold nanoparticles (Au NPs) offer a highly versatile platform for brain‐targeted drug delivery due to their small size, excellent colloidal stability, and tunable surface chemistry.^[^ ^12^ ^]^ Unlike conventional delivery systems such as liposomes or polymeric NPs, Au NPs easily allow precise control over size, charge, and ligand presentation, enabling efficient cellular uptake and intracellular delivery.^[^ ^13^ ^]^ Moreover, the inert gold core permits chemical stability and offers potential for theranostic integration, including computed tomography (CT) or photoacoustic imaging and photothermal therapy.^[^ ^14^ ^]^ These advantages, along with a growing body of preclinical safety ansigd pharmacokinetic data, support the use of Au NPs as an ideal nanocarrier for redox‐modulating therapies such as glutathione delivery in brain cancer.^[^ ^15^ ^]^

Despite the promising results, GSH delivery to the central nervous system (CNS) is hampered by its inherent instability and low bioavailability. In this regard, conjugation with biomolecules able to facilitate BBB‐crossing has been shown to improve drug absorption.^[^ ^16^ ^]^ We already demonstrated that functionalization with galactose molecules could facilitate internalization by targeting the GLUT transporter, which is overexpressed on the BBB.^[^ ^17^ ^]^ In this study, we exploited a similar strategy to develop a smart gold nanoparticle (Au NP)‐based system featuring galactose‐mediated increased GSH internalization efficiency. This system aims to mitigate oxidative stress by exploiting GSH molecules bound to the surface of Au NPs and its antioxidant performance was evaluated in brain cancer cell models.

## 2 Results and Discussion

Au NPs have been extensively used and explored for biomedical applications owing to their unique physical, chemical, and biological properties, which confer remarkable versatility across a spectrum of applications from drug delivery to diagnostic. Their high surface area to volume ratio, ease of functionalization, and biocompatibility render them ideal candidates for targeted drug delivery systems, thereby enhancing the efficacy of therapeutic agents while minimizing side effects.^[^ ^18^ ^]^

### 2.1 Synthesis and Characterization of the Au@GSH‐Gal NPs

**Scheme 1:**
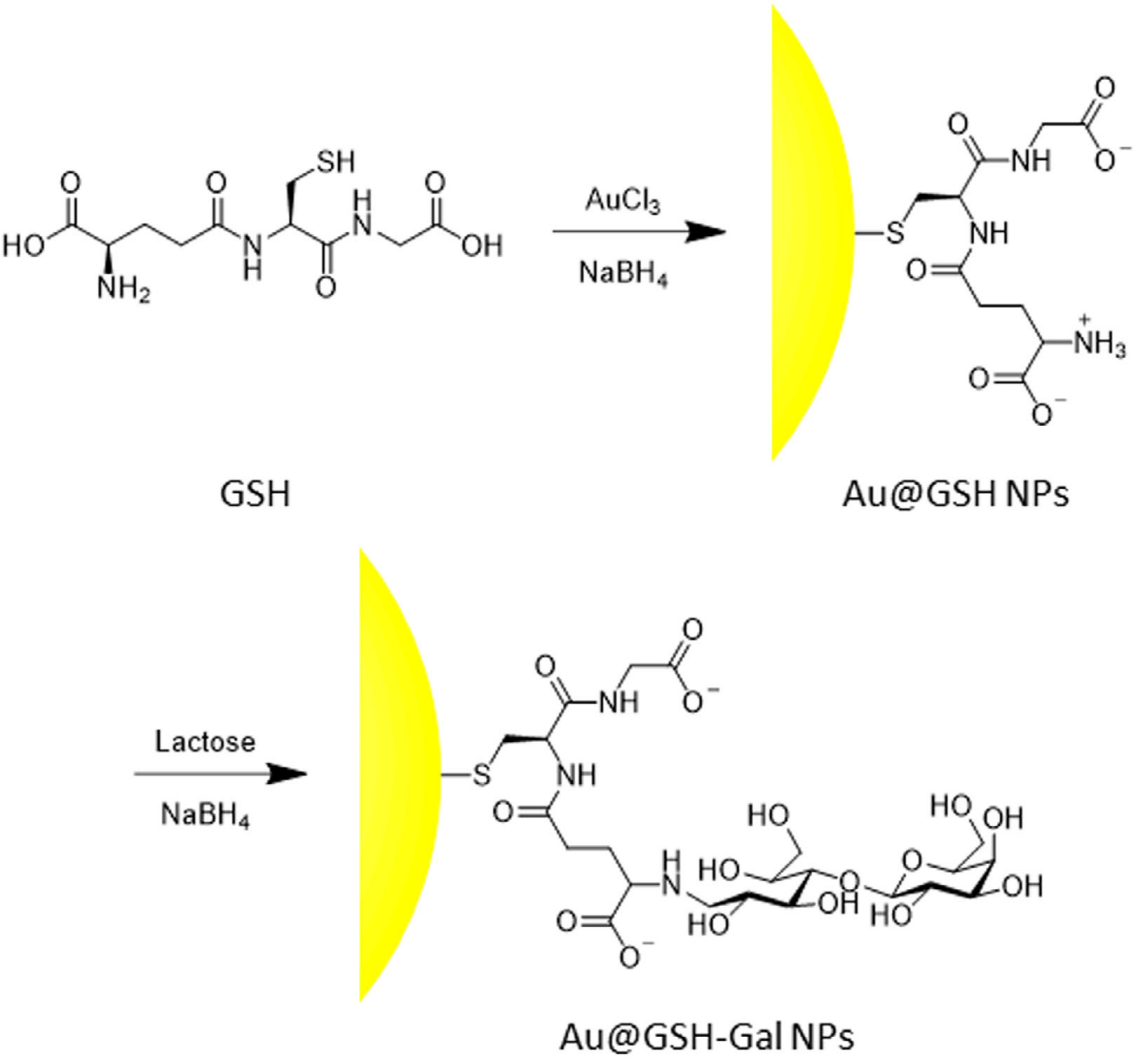
Scheme of the synthetic steps utilized to prepare the Au@GSH‐Gal NPs.

In this study, a straightforward gold reduction in a thiol‐containing solution was exploited for the synthesis of Au@GSH NPs,^[^ ^19^ ^]^ exploiting the cysteine's thiol group for passivating the surface of the NP. Subsequent reaction between the free amino group of the glutamic acid of GSH and the reducing end of the glucose moiety of lactose, followed by reduction of the formed imine, yielded a secondary amine linked to a polyol spacer exposing a galactose residue (**Scheme** **1**). A similar strategy had already been successfully implemented by our group for targeting the GLUT transporter using galactose‐ and dopamine‐functionalized CdSe/CdS quantum rods, proving its validity and efficacy.^[^ ^17^ ^]^ We initially explored the reductive amination step at various concentrations (data not shown). However, the NPs exhibiting near‐complete functionalization demonstrated the highest stability and were therefore selected for use in this study.

**Figure 1:**
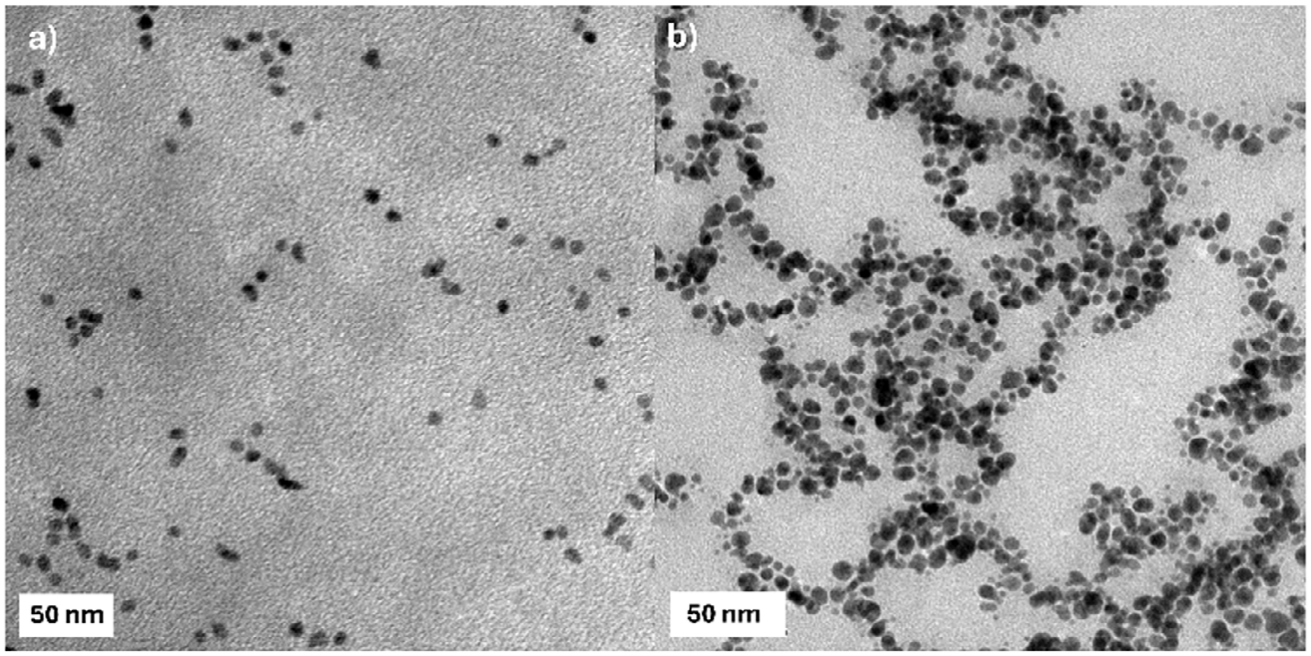
TEM images of the a) Au@GSH and b)Au@GSH‐Gal NPs. Scale bar is 50 nm.

Following dialysis, transmission electron microscopy (TEM) imaging revealed that the Au@GSH NPs exhibited a high degree of monodispersity and possessed a spherical inorganic core with an average diameter of 5.5 ± 0.9 nm (**Table** **1** and **Figure** **1**a).

**Table 1:** Physicochemical characterization of the NPs as determined by TEM and DLS analyses in H_2_O (pH 7.0).

NPs	Core diameter [nm]	Hydrodynamic diameter [nm]	PDI	ζ‐Potential [mV]
Au@GSH	5.5 ± 0.9	10.7 ± 0.5	0.20	−21.7 ± 2.3
Au@GSH‐Gal	7.8 ± 1.8	24.2 ± 3.8	0.24	−52.0 ± 1.0

Following reductive amination, the average size of the inorganic core increased to 7.8 ± 1.8 nm and a slightly more polydisperse distribution could be observed under the electron microscope (Figure 1b).

Dynamic light scattering (DLS) analysis in aqueous solution yielded a hydrodynamic diameter of ≈11 nm for the GSH‐coated NPs, a value consistent with the dimensions observed via TEM (Table 1). In contrast, the hydrodynamic size of the Au@GSH‐Gal NPs was ≈24 nm, roughly 3 times larger than the inorganic core diameter. This substantial increase is likely attributable not only to the additional galactose‐containing layer on the nanoparticle surface, which would be expected to augment the size by a few nanometers, but also to the formation of noncovalent interactions among the carbohydrate‐decorated NPs. This hypothesis is further supported by the slight increase in the polydispersity index (PDI) from 0.2 for the Au@GSH NPs to 0.24 for the Au@GSH‐Gal NPs. The size distribution profiles for both types of NPs exhibited well‐defined unimodal curves (Figure S1, Supporting Information). Nevertheless, both Au@GSH and Au@GSH‐Gal NPs demonstrated excellent dispersibility in aqueous media, with no evidence of precipitation observed either before or after functionalization.

Consistent with the presence of carboxylate ions on the GSH ligand, zeta potential measurements revealed a negative surface charge of about −22 mV for the Au@GSH NPs. This value suggests good colloidal stability in aqueous media due to electrostatic repulsion.^[^ ^20^ ^]^ In addition, the subsequent reductive amination step, by masking the terminal amino group on the glutathione residue, resulted in an approximate doubling of the negative surface charge to about −52 mV.

The UV‐Vis absorption spectra of the purified NPs evidenced a maximum peak at 538 and at 523 nm for the Au@GSH and Au@GSH‐Gal NPs, respectively. The observed blueshift could be ascribed to the formation of soluble clusters with reduced mobility, consistent with the findings from the DLS measurements (Figure S2, Supporting Information).^[^ ^21^ ^]^

The Fourier transform infrared (FT‐IR) spectrum of the Au@GSH NPs between 600 and 1750 cm^−1^ displayed the characteristic bands of the GSH molecule, while the Au@GSH‐Gal NPs revealed the appearance of a major broad peak centered at 1080 cm^−1^, analogous to that observed for the free carbohydrate, further corroborating the successful functionalization (Figure S3, Supporting Information).

**Figure 2:**
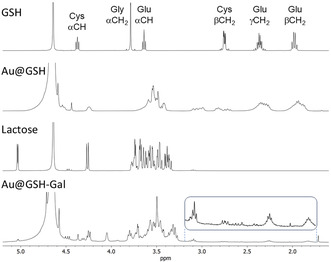
^1^H NMR spectra in D_2_O of (from top to bottom) free GSH, the Au@GSH NPs, lactose, and the Au@GSH‐Gal NPs.

The ^1^H nuclear magnetic resonance (NMR) spectra of the NPs were recorded in deuterated water (D_2_O) at 400 MHz to elucidate the chemical composition of their outer shell (**Figure** **2**).

All major resonances characteristic of GSH were also identified in the ^1^H NMR spectrum of the Au NPs‐conjugated GSH. As expected, additional splitting of the signals, increased multiplicity, and signal broadening were observed as a result of the decreased mobility of the molecules upon binding to the NP, and consequently longer *T*_2_ relaxation times, thus freezing the ligands in a more rigid conformation. This effect is particularly pronounced in the signals corresponding to the methylene protons directly bonded to the thiol moiety, which exhibited a downfield shift upon binding to the gold surface. In addition, these same hydrogens, which are diastereotopic but chemically equivalent in the freely rotating GSH molecule (yielding a doublet at 2.70 ppm), resolved into two distinct multiplets at 3.20 and 2.91 ppm when the molecule was bound via the thiol to the NP surface, a spectral feature also observed in the oxidized GSSG dimer.^[^ ^22^ ^]^ Similarly, the two ‐CH_2_‐ of the glutamate side chain in alfa and beta to the amide bond with cysteine transitioned from a triplet and a quartet to multiplets. On the other hand, the alfa hydrogens of cysteine, initially appearing as a triplet, broadened into a singlet due to the reduced mobility.

The reductive amination of lactose with the free amine group of the Au@GSH NPs led to the formation of a polyol chain terminating in a galactose residue. The success of this reaction was confirmed by the NMR spectrum, specifically by the appearance of two triplets at about 3.1 and 3.3 ppm, which assessed the formation of two diastereotopic protons in alpha to the secondary amine generated by the reductive amination step. Furthermore, only the resonances corresponding to the outer shell of sugar were easily observed, while those corresponding to the GSH linker were discernible only upon significant amplification of the signal intensity (see the inlet in Figure 2).

The functionalization of the amino groups on the surface of the Au@GSH NPs was further confirmed by a quantitative Kaiser test, which evidenced the near‐complete disappearance of the characteristic fluorescent adduct peak associated with the primary amine of the glutamic acid residue following functionalization with galactose (Figure S4, Supporting Information).^[^ ^23^ ^]^ Utilizing a calibration curve generated with free GSH and normalizing the interpolated value to the concentration of Au NPs (determined by optical measurement), an average number of ≈5500 GSH molecules bound per nanoparticle was estimated in the Au@GSH NPs. This experimentally derived value exceeds theoretical calculations. In fact, assuming an average core diameter of 5.5 nm, as determined by TEM analysis, a hypothetical spherical surface area of about 99 nm^2^ and a Gaussian curvature (*K*) of about 0.13 nm^2^ can be calculated. Considering a molecular footprint of about 0.2 nm^2^ for each GSH molecule, as reported in the literature, ≈495 bound molecules per nanoparticle would be theoretically expected.^[^ ^24^ ^]^ This discrepancy may be attributed to the presence of additional adsorbed molecules forming a thick organic layer around the inorganic core. Comparing the Kaiser test results before and after reductive amination allowed for a rough estimation of ≈986 free amine groups per NP in the Au@GSH NPs, suggesting ≈4510 galactose units per NP in the Au@GSH‐Gal NPs.

Elemental analysis using inductively coupled plasma atomic emission spectrometry (ICP‐AES) was also performed to estimate the S/Au and S/Au NP molar ratios. The results, reported in Table S1, Supporting Information, indicated S/Au NP ratio of 3335 ± 519, a value that correlates favorably with the estimate obtained by the Kaiser test, given the one‐to‐one correspondence between each sulfur atom and a GSH molecule. The S/Au molar ratio was found to be 0.62 ± 0.10.

Finally, the electrophoretic migration of the NPs as a function of their mass and charge was investigated by gel electrophoresis (Figure S5, Supporting Information), corroborating the findings previously highlighted by the DLS measurements. Specifically, the Au@GSH NPs exhibited a distinct band, indicative of a homogeneous population within the sample, while, following conjugation with galactose, a slightly broadened band was observed on the gel, suggesting a greater heterogeneity in the size and/or charge of the NPs population. Notably, the migration of the Au@GSH‐Gal NPs was also slower than that of the Au@GSH NPs, attributable to alterations in both size and surface charge. The electrophoretic migration, along with the other characterization analyses, was assessed using aliquots from different preparations, evidencing the high reproducibility of both the synthesis and functionalization procedures.

### 2.2 Antioxidant Assays

**Figure 3:**
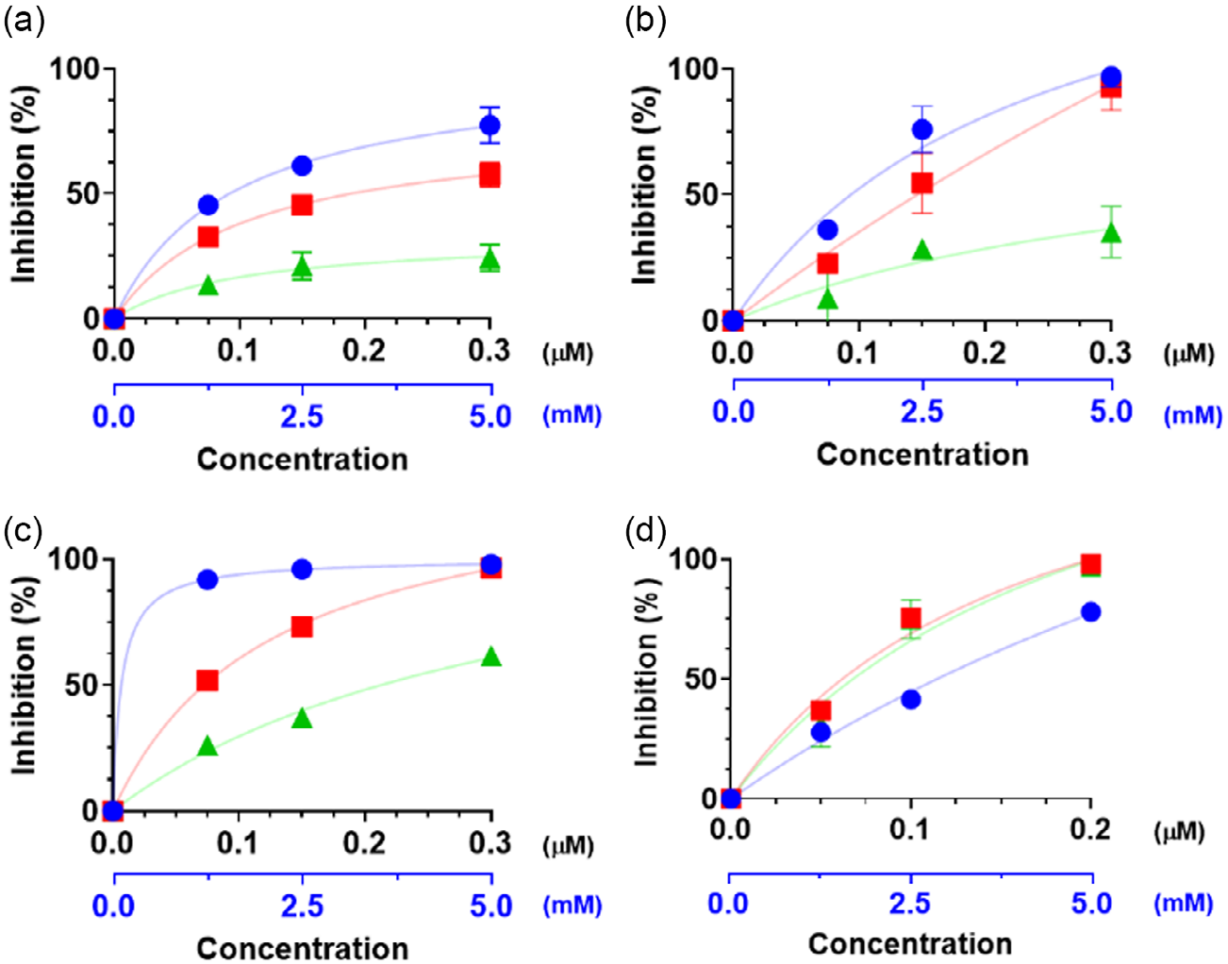
Radical scavenging activity of free GSH (blue dots), Au@GSH, (red squares), and Au@GSH‐Gal NPs (green triangles) at three different concentrations according to the a) DPPH, b) TEAC, c) FRAP, and d) hydrogen peroxide assays. The black *x*‐axis refers to the concentrations of the NPs; the blue *x*‐axis refers to the concentrations of free GSH.

Preliminary chemical analyses were performed to evaluate the antioxidant capacity of the systems using different assays, namely 2,2‐diphenyl‐1‐picrylhydrazyl (DPPH), trolox equivalent antioxidant capacity (TEAC), ferric ion reducing antioxidant power (FRAP), and hydrogen peroxide assay (**Figure** **3**). In general, the Au@GSH NPs demonstrated an antioxidant effect, albeit typically to a slightly lesser degree compared to the corresponding estimated concentration of free GSH. This observation is not unexpected and could be attributed to the sterically hindered antioxidant activity of the GSH molecule, and consequently its thiol group, when bound to the NP surface.

As expected, a direct positive correlation was observed between the antioxidant activity and increasing concentrations of both the NPs and the free ligand. A modest reduction in the antioxidant efficacy of the NPs was frequently noted following conjugation with galactose. While seemingly counterproductive, this is plausible as the GSH molecules are now partially sterically hindered by the presence of the targeting galactose moiety. Nevertheless, this marginal decrease in efficiency is not anticipated to be a significant concern, as galactose is expected to substantially enhance the targeting specificity and cellular internalization, thereby rendering the antioxidant effect of the galactose‐functionalized NPs considerably more effective compared to their galactose‐free counterparts. Furthermore, it is anticipated that the NPs will undergo degradation postinternalization, leading to the release of the GSH ligands and the recovery of their full antioxidant potential intracellularly.^[^ ^25^ ^]^ As an additional control, the radical scavenging activity of free galactose was assessed at concentrations corresponding to those estimated on the NP surface, but no significant effect was detected in the 0.15–1.5 mM range (data not shown).

The antioxidant capacity of the synthesized NPs was further evaluated by measuring their ability to scavenge a fixed concentration of hydrogen peroxide, a well‐established source of radicals. Both Au@GSH and Au@GSH‐Gal NPs were tested to investigate if the terminal galactose moiety would hinder the antioxidant capacity of the NP‐bound GSH. Three increasing concentrations of NPs, that is, 0.05, 0.10, and 0.20 mM, were examined to elucidate the concentration‐dependent antioxidant relationship. Free GSH was also assessed to provide a quantitative benchmark for the scavenging efficacy of the NPs, showing to be able to inhibit about 28% of the added hydrogen peroxide at the lowest concentration tested (1 mM), a slightly greater amount (about 41%, *p* > 0.05) at 2.5 mM GSH, and about 78% (*p* < 0.0001) at the highest GSH concentration (5 mM) (Figure 3d). The percentage of inhibition ranged from about 37% to 31% at the lowest concentration (0.05 mM) tested of Au@GSH and Au@GSH‐Gal NPs, respectively, comparable to that observed with 1 mM free GSH. The inhibitory effect increased significantly with increasing concentrations of NPs, reaching values of about 75% and 98% at 0.1 and 0.2 mM concentrations, respectively. In general, the difference in inhibition was more statistically significant when transitioning from the lowest to the intermediate concentration (*p* < 0.005), while the increase was less pronounced when shifting from the intermediate to the highest concentration (*p* < 0.01). Notably, in contrast to the other antioxidant assays, no significant difference in ROS scavenging was observed between the Au@GSH and the Au@GSH‐Gal NPs at the same concentration.

### 2.3 Cellular Studies

Prior to conducting any antioxidative assays on cells, the biocompatibility of the synthesized NPs was evaluated at various concentrations following a 24 h incubation period. Human neuroblastoma (SH‐SY5Y) and rat glioblastoma (C6) cells were employed as in vitro models for these experiments due to their established relevance to oxidative stress and neurodegenerative processes. Notably, both cell lines are reported to express glucose transporters.^[^ ^26^ ^]^

**Figure 4:**
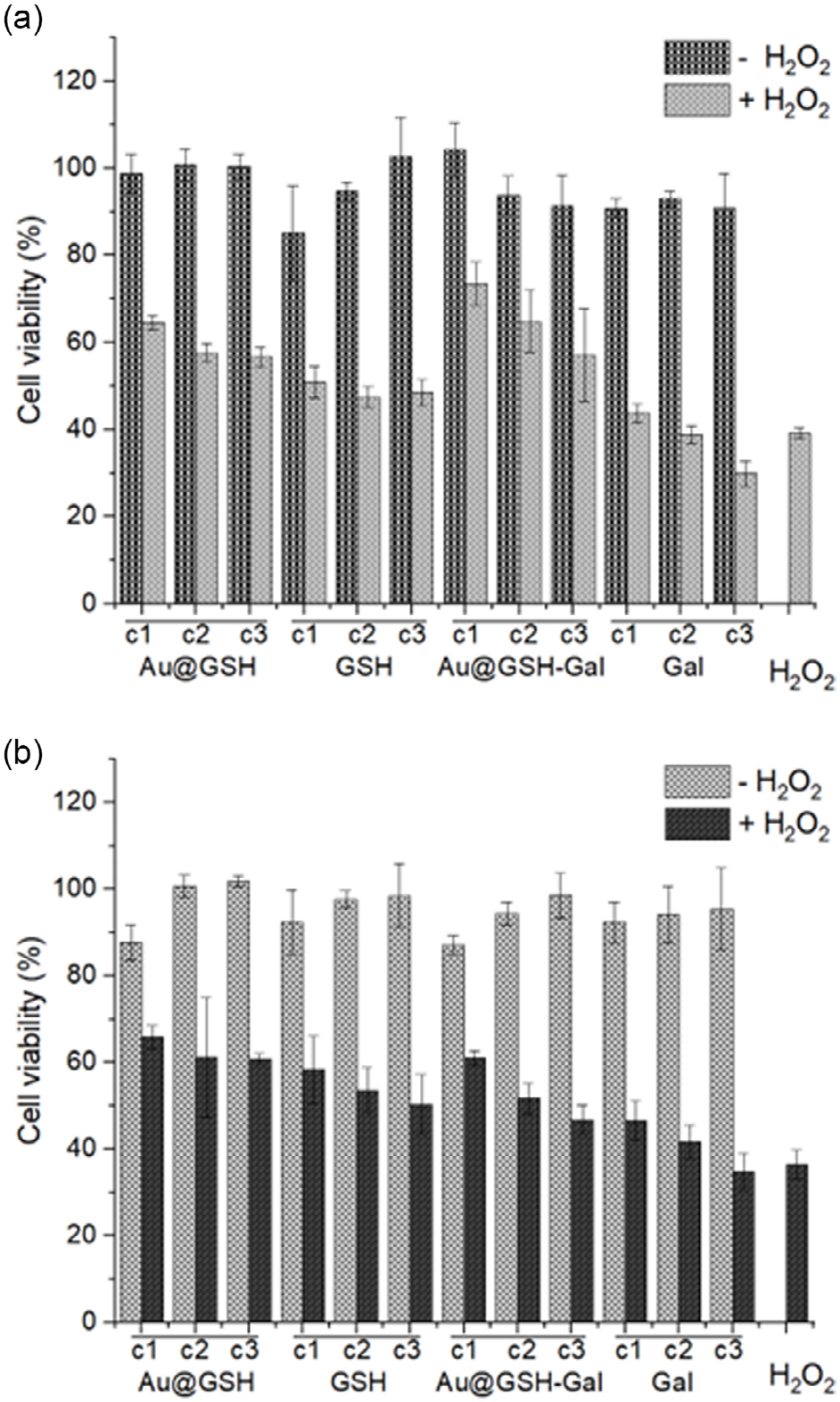
MTT assay performed with a) SH‐SY5Y and b) C6 cells incubated with Au@GSH, free GSH, Au@GSH‐Gal, and free Gal for 24 h at three different concentrations (c1, c2, and c3 correspond to 100, 50, and 25 nM NP concentration; 500, 250, and 125 μM in the case of free GSH; and 100, 50, and 25 μM in the case of free Gal, respectively). The assay was also performed upon addition of 2 mM hydrogen peroxide for 1 h, after the NP incubation.

The thiazolyl blue tetrazolium bromide (MTT) colorimetric assay was initially performed to evaluate cell viability following the administration of the free ligands (GSH and Gal) and the corresponding NPs. Excellent biocompatibility was observed for all NPs and their respective controls across the tested concentration range (**Figure** **4**). The free GSH and galactose molecules were also assessed across a range of concentrations equivalent to the approximate amount of free ligand present on the corresponding NPs. Three distinct concentrations (c1, c2, and c3, corresponding to 100, 50, and 25 nM NPs concentration, 500, 250, and 125 μM in the case of free GSH, and 100, 50, and 25 μM in the case of free Gal, respectively) were evaluated.

As shown in Figure 4, the viability of both SH‐SY5Y and C6 cells following incubation with Au@GSH and Au@GSH‐Gal NPs was comparable to that of the untreated control cells. A marginal reduction in viability (≈87%) was observed only in C6 cells exposed to the highest concentration (100 nM) of both types of NPs. These findings underscore the excellent biocompatibility of the NPs under the tested conditions. Furthermore, the cells were also exposed to the equivalent concentrations of free GSH and Gal, which, as expectable, resulted in ≈100% viability.

Upon the addition of 2 mM hydrogen peroxide to the cell culture medium, the viability of the control cells dropped below 37% in both SH‐SY5Y and C6 cell lines. On the other hand, when the cells were pre‐incubated with the NPs, regardless of whether they were functionalized with GSH or GSH‐Gal, a protective effect against H_2_O_2_‐induced cytotoxicity was observed, with viability remaining above 50% in all cases and at all tested concentrations. Moreover, this protective effect exhibited a concentration‐dependent relationship, increasing with higher NPs concentrations and, consequently, greater amounts of GSH supplied. A similar protective effect was noted with free GSH, whereas free Gal did not confer any significant protection, with cell viability comparable to that of cells directly exposed to oxidative stress.

To further substantiate the antioxidant activity of the NP‐anchored GSH, the 2′,7′‐dichlorofluorescein diacetate (DCF) assay was performed.

**Figure 5:**
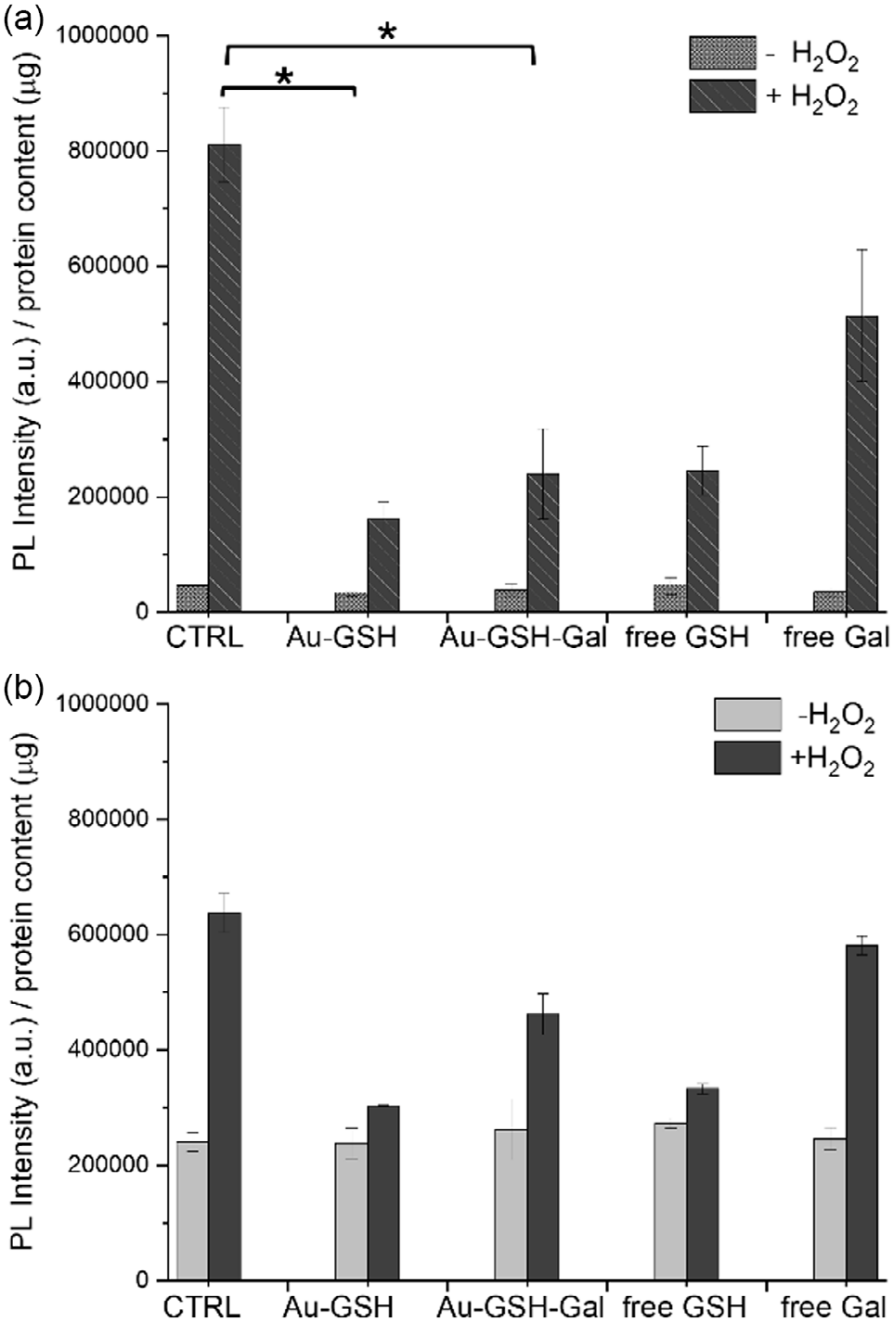
DCF assay performed with a) SH‐SY5Y and b) C6 cells incubated with Au@GSH, free GSH, Au@GSH‐Gal, and free Gal for 24 h at 100 nM NP concentration (that corresponds to 500 μM free GSH and 100 μM free Gal, respectively). To induce an oxidative stress, hydrogen peroxide (2 mM) was added to the cells for 1 h, after the incubation with the NP (* corresponds to *p* < 0.05).

The cells were incubated with the NPs (100 nM, equivalent to 500 μM GSH) for 24 h prior to being exposed to 2 mM hydrogen peroxide for 1 h. The histograms in **Figure** **5** show the results of the assay performed with both cell lines and presented as the average photoluminescence (PL) intensity normalized over the protein content. The data clearly indicate that the addition of H_2_O_2_ to the control cells triggered the production and release of ROS species. On the other hand, when the cells were pretreated with the NPs, the detected ROS levels were significantly lower. Interestingly, the protective effect of the NPs was observed even when they were functionalized with galactose. Pretreatment with free GSH also provided substantial protection against ROS generation. As a general observation, although a direct quantitative comparison is not feasible, the nanoparticle‐mediated radical scavenging effect appeared to be more pronounced in the neuroblastoma cells, where the ROS levels in nanoparticle‐treated cells exhibited a significant reduction compared to the H_2_O_2_‐treated control samples.

Indeed, the ratio of normalized ROS signal in control cells treated with H_2_O_2_ to that in cells treated with Au@GSH and H_2_O_2_ was 5 in SH‐SY5Y cells, whereas this ratio was 2 in glioblastoma cells.

**Figure 6:**
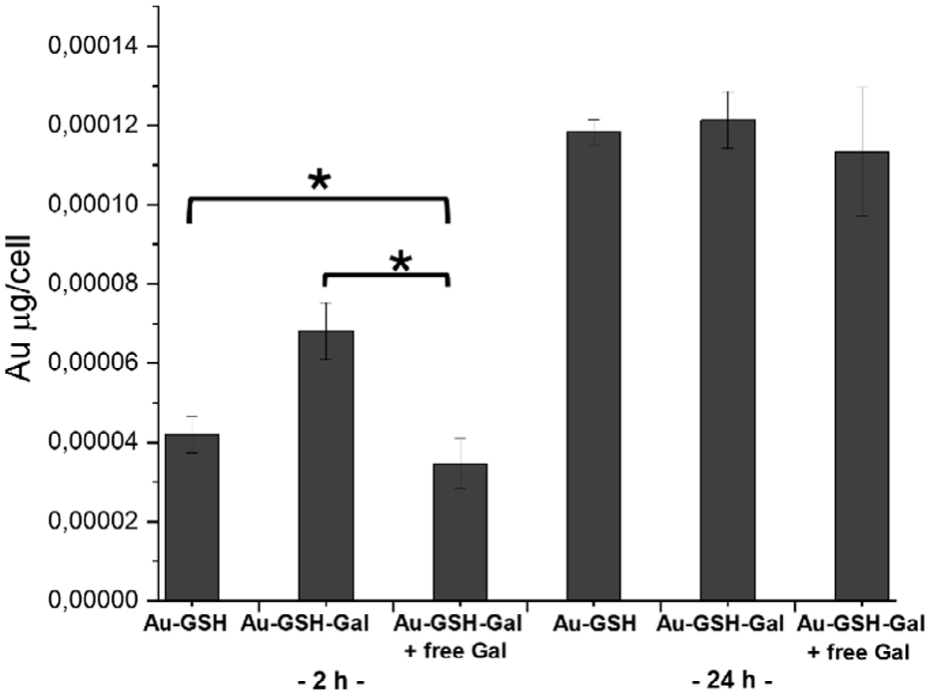
Cellular uptake of Au NPs, as estimated by ICP elemental analysis. SH‐SY5Y cells were incubated with either Au@GSH or Au@GSH‐Gal for 2 and 24 h. A competitive condition was included by preincubating cells with 1 mM free Gal for 30 min prior to administering the NPs. The mass of gold was normalized over the number of cells (* corresponds to *p* < 0.05).

To elucidate whether surface functionalization with galactose enhances the internalization process of the NPs, elemental analysis of SH‐SY5Y cells treated with either Au@GSH or Au@GSH‐Gal for 2 and 24 h was performed. The intracellular nanoparticle content, expressed as Au (μg)/cell, revealed that after 2 h of incubation, cells treated with Au@GSH‐Gal had internalized a greater quantity of NPs compared to cells exposed to Au@GSH (**Figure** **6**). Interestingly, when cells were competitively pretreated with 1 mM galactose for 30 min to saturate membrane receptor units, the amount of Au detected was significantly lower compared to that in cells treated with Au@GSH and Au@GSH‐Gal NPs. This finding strongly suggests the involvement of galactose transporters in the recognition and internalization of the Au@GSH‐Gal NPs.

Furthermore, extending the incubation period to 24 h resulted in an increase in the intracellular amount of Au/cell from 4 × 10^−5^ and 6.8 × 10^−5^ μg for Au@GSH and Au@GSH‐Gal, respectively, to 1.1 × 10^−4^ and 1.2 × 10^−4^ μg, respectively. Notably, no significant difference in intracellular gold levels was observed between the two NP treatments at the 24 h time point, suggesting the potential involvement of passive internalization mechanisms.

**Figure 7:**
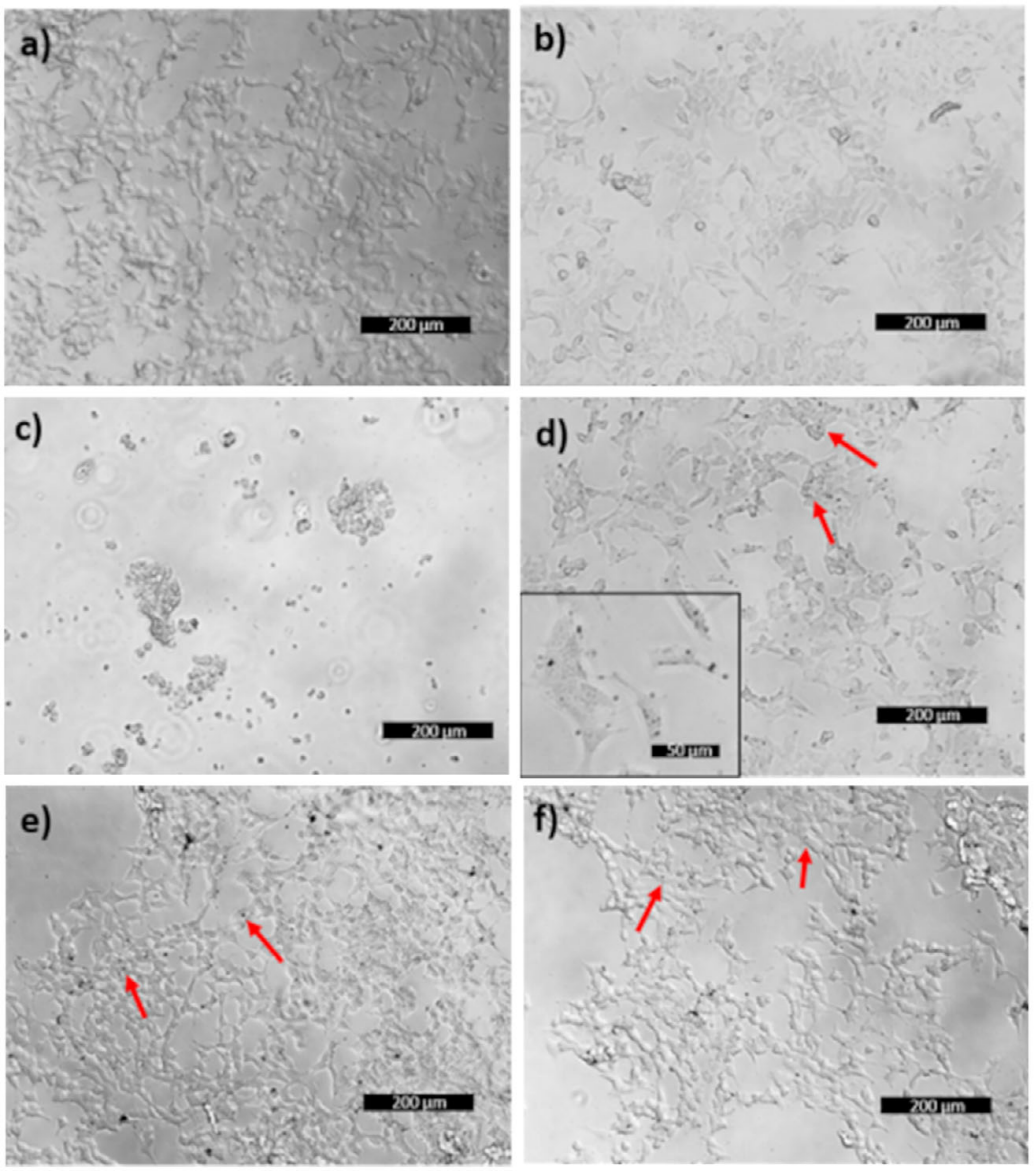
Representative optical images of SH‐SY5Y cells incubated with either a) the vehicle, b) free GSH, c) free Gal, d) Au@GSH, e) Au@GSH‐Gal, or f) Au@GSH‐Gal + free Gal, for 24 h. The red arrows indicate the cell endosomes containing the internalized NPs. The scale bar corresponds to 200 μm.

The cellular internalization of Au NPs was further corroborated by optical imaging and ultrastructural analysis of cell samples. The images of **Figure** **7** show a) control SH‐SY5Y cells, b) cells incubated for 24 h with GSH, c) cells treated with H_2_O_2_, d) cells treated with Au@GSH, e) cells treated with Au@GSH‐Gal, and f) cells treated with Au@GSH‐Gal in the presence of free galactose. The addition of hydrogen peroxide induced cellular stress and death, as evidenced by the presence of rounded and detaching cells (Figure 7c).

Intracellular endosomes containing the NPs (Figure 7d–f) were identifiable by the presence of dark spots localized within cells displaying a regular morphology and distribution across the culture plate, consistent with the control sample.

**Figure 8:**
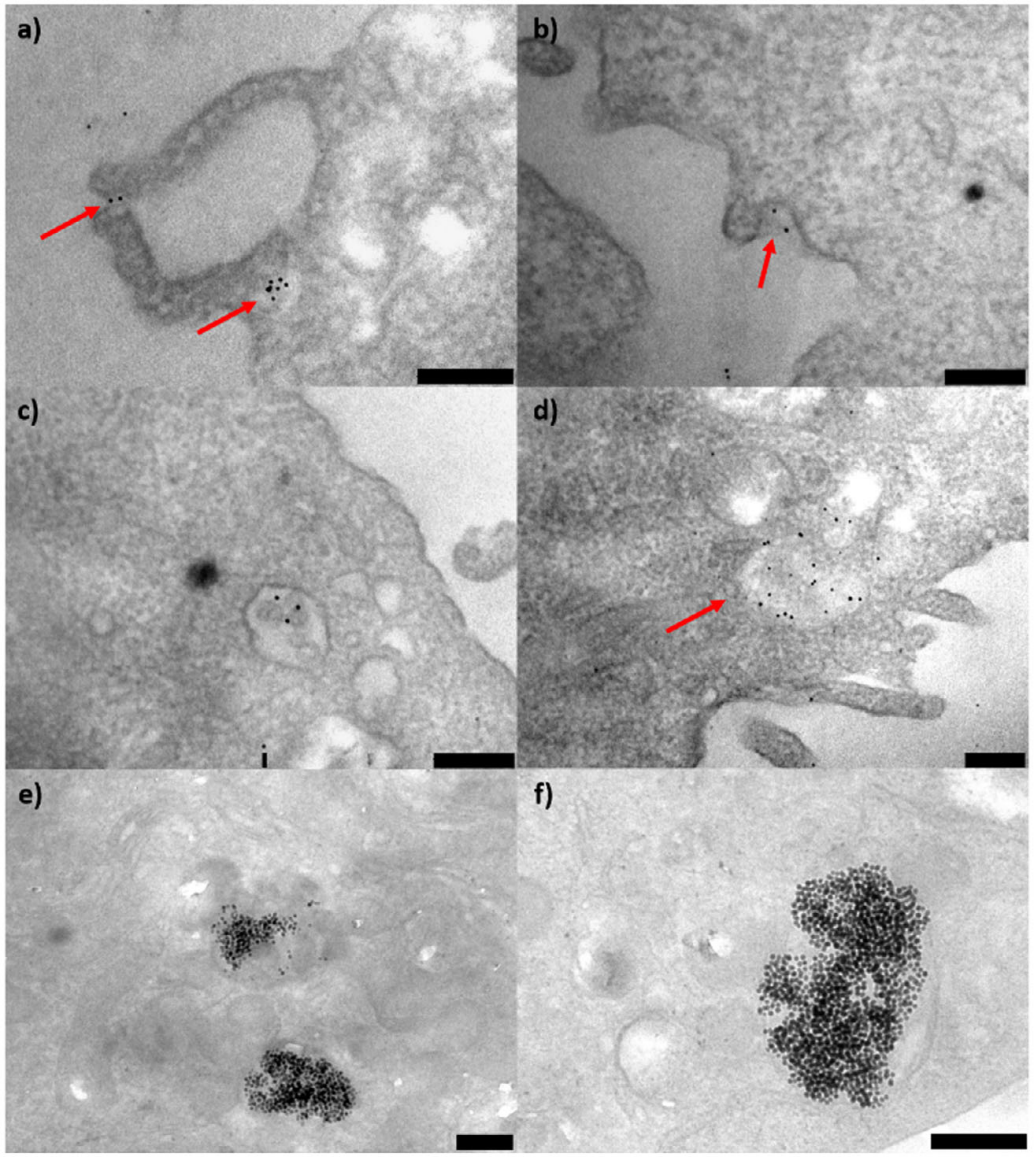
TEM images of SH‐SY5Y cells incubated for a–d) 2 h and e,f) 24 h with Au@GSH NPs, respectively. The red arrows indicate the engulfment process and the early endosomes containing the NPs. The scale bar corresponds to 250 nm.

The ultrastructural analysis (**Figure** **8**) of cells incubated with Au@GSH NPs for 2 and 24 h illustrates the temporal progression of nanoparticle internalization and intracellular compartmentalization. Specifically, at the 2 h time point, NPs in close proximity to the cell membrane and encapsulated within early endosomes were observed (Figure 8a–d). In contrast, after 24 h, the predominant feature was the presence of large endosomes containing numerous NPs.

## 3 Conclusion

Due to its antioxidant properties and ability to cross the BBB, GSH has been extensively investigated both as a potent natural antioxidant and as a transport mediator for brain drug delivery.^[^ ^27^ ^]^ While its use has been known for many years, recent studies focus on GSH exploitation in nanoformulations and nanocarriers. For instance, GSH‐loaded solid–lipid NP‐enriched hydrogel has been developed for skin penetration and topical sustained release in antiaging applications.^[^ ^28^ ^]^ It has been shown that GSH can also act as a shuttle across the BBB to enhance the diffusion of inorganic or organic NPs for controlled drug delivery. Examples include magnetic NPs conjugated to GSH, which exploited GSH‐mediated transport to brain tumors for MR imaging and paclitaxel delivery.^[^ ^29^ ^]^ GSH‐coated polymeric NPs and pegylated liposomes have also been developed and applied for CNS delivery of docetaxel and methylprednisolone, respectively.^[^ ^30^ ^]^ Several studies have shown that the presence of GSH significantly increases the cellular uptake of liposomes across the BBB.[^30^, ^31^] Notably, while polymeric and lipid‐based NPs offer a hosting compartment for drugs, they may face limitations in delivery efficacy due to size constraints. On the other hand, inorganic NPs, generally with sizes below 10 nm, can benefit from enhanced transport and rapid in vivo kinetics, potentially overcoming safety concerns related to toxicity and bioaccumulation. In addition, their inorganic domain enables their use as imaging probes, as demonstrated in pioneering studies using ultrasmall GSH‐capped and radio‐labeled Au NPs as single‐photon emission computed tomography (SPECT) and near‐infrared (NIR) imaging tools.^[^ ^32^ ^]^ Thus, the presence of GSH on the surface of Au NPs provides additional features to the overall structure, enabling its potential use as both antioxidant and imaging system.^[^ ^33^ ^]^

In our study, small GSH‐capped Au NPs were readily prepared using a straightforward one‐step procedure. GSH serves to provide optimal stability and biocompatibility to the NPs while maintaining its inherent antioxidant property. The imaging potential of these NPs will be investigated in future studies. The GSH‐bound Au NPs were further functionalized with galactose on the outer shell to target the GLUT transporter, which is overexpressed on the BBB. The resulting Au@GSH‐Gal NPs were characterized in detail and demonstrated significant radical scavenging activity in vitro. In addition, they effectively reduced oxidative stress in both neuroblastoma and rat glioma cells. Competitive experiments with free galactose suggest specific internalization by targeting membrane transporters, as previously demonstrated,^[^ ^17^ ^]^ while optical imaging and TEM structural analyses revealed the formation of endosomes containing increasing amount of gold NPs over time. Overall, these findings indicate that the Au@GSH‐Gal NPs possess optimal stability and biocompatibility while retaining the inherent antioxidant property of the GSH molecule, and the imaging potential of these NPs will be explored in future studies.

## 4 Experimental Section

4.1

4.1.1

##### Chemicals

Gold (III) chloride (AuCl_3_ 99%, #334 049), glutathione (C_6_H_17_NO_6_S 98%, #G4251), sodium borohydride (NaBH_4_ 98%, #452 882), lactose (C_12_H_22_O_11_ 99%, #L3750), 1,1‐diphenyl‐2‐picrylhydrazyl (DPPH), hydrogen peroxide (H_2_O_2_), Triton X‐100, and ferric chloride (FeCl_3_) were purchased from Sigma–Aldrich. Solvents were used without further purification. Ultrapure water was used in all steps.

##### Synthesis of the Au@GSH‐Gal NPs

The NPs were prepared following the Brust method.^[^ ^19^ ^]^ Briefly, a solution of AuCl_3_ (0.012 g, 0.04 mmol) dissolved in H_2_O (1.6 mL) was added to a solution of GSH (0.123 g, 0.4 mmol) in H_2_O (33 mL). Then, NaBH_4_ (0.030 g, 0.8 mmol) was dissolved in H_2_O (0.8 mL) and poured into the vial containing the gold solution. The mixture was left to react for 2 h under slow mechanical stirring at 9 rcf and then washed three times with ultrapure water using Amicon filters (10 kDa MWCO) at 1200 rcf for 30 min. Finally, the solution was filtered over cellulose filters (0.2 μm MWCO) to eliminate any aggregates and obtain a homogeneous solution consisting of Au@GSH NPs. Subsequently, lactose (2.7 mg, 8 μmol) was dissolved in phosphate‐buffered saline (PBS, pH 7.4, 0.3 mL) and added to an aqueous solution of Au@GSH NPs (0.5 mL, 1 μM). Sodium borohydride (NaBH_4_, 0.45 mg, 12 μmol) was dissolved in PBS (pH 7.4, 0.2 mL) and slowly added to the previous solution. The mixture was shaken at 9 rcf for 2 h and then washed three times with ultrapure water using Amicon filters (10 kDa MWCO) at 1200 rcf for 30 min. After purification, the NPs were dissolved in H_2_O to a final concentration of 1 μM and used as such for the subsequent characterization and in vitro experiments.

##### Characterization of the Au@GSH NPs

TEM analysis was performed with a JEOL Jem1011 microscope operating at an accelerating voltage of 100 kV. A drop of the solution containing the NPs was deposited onto carbon‐coated copper grids (Formvar/Carbon 300 Mesh Cu) and left to dry before analysis. The images obtained from the TEM analysis were examined using the ImageJ software (https://imagej.net/ij/index.html) which produces as a result of the statistical analysis of the sample an estimation of the NPs diameter and of other morphological characteristics such as circularity, aspect ratio and homogeneity.

DLS analyses were performed with a Zeta Sizer Nano ZS90 instrument (Malvern Instruments Ltd., Malvern, UK) with 40 mW He‐Ne laser, operating at 633 nm and an APD photodiode detector. The measurements were carried out at 25 °C and are reported as average ± standard deviation of at least three replicates for each sample. The samples were dissolved in ultrapure water.

The UV‐Vis absorption spectra were recorded at 25 °C between 350 and 800 nm by using a Varian Cary 300 UV‐Vis instrument and quartz cuvettes with 1 cm light path.

Fourier transform infrared (FT‐IR) spectra were acquired by ATR with a Perkin Elmer Spectrum One Fourier Transform spectrophotometer (Waltham, MA, USA) set up with 64 scans and a resolution of 4 cm^−1^.

The electrophoretic mobility of the NPs was carried out on 1% agarose gel in Tris‐Borate‐EDTA (TBE) buffer. To prepare the gel, 0.5 g of agarose was dissolved in 50 mL of 0.5% TBE. The run was performed at 100 V for 30 min.

^1^H NMR spectra were recorded in D_2_O at 25 °C on a Varian Mercury operating at 400 MHz. Chemical shifts are reported in ppm and the spectra were referenced to the solvent peak.

The DPPH, TEAC, FRAP, and hydrogen peroxide antioxidant assays were performed as already described.^[^ ^34^ ^]^

##### Biological Studies

Two cell lines, the human SH‐SY5Y neuroblastoma and the rat C6 glioblastoma, were supplied by ATCC. Both types of cells were grown in DMEM medium supplemented with 10% fetal bovine serum (FBS), 2 mM glutamine, 100 IU mL^−1^ of penicillin, and 100 μg mL^−1^ of streptomycin in an incubator at 37 °C in a humidified atmosphere with 5% CO_2_.

The cytocompatibility of the Au NPs and their protective effect against oxidative stress were evaluated using the MTT assay. The cells (either SH‐SY5Y or C6 cells) were seeded in 96‐well plates at a density of 2.5 × 10^4^ per well and incubated at 37 °C in 5% CO_2_. The medium was then replaced with fresh medium containing either Au@GSH (100, 50, and 25 nM), Au@GSH‐Gal (100, 50, and 25 nM), free GSH (500, 250, and 125 μM), or free Gal (100, 50, and 25 μM), and the cells were kept under incubation at 37 °C for 24 h. Hydrogen peroxide (2 mM) was then added to the wells for 1 h to trigger oxidative stress. After that, the medium was removed and replaced with a serum‐free medium containing 2 mg mL^−1^ MTT and incubated for 2 h at 37 °C. The MTT reagent was then removed and the formazan crystals were solubilized using dimethyl sulfoxide. The absorbance (at 570 nm) was read using the CLARIO star Plus microplate reader. The absorbance of the vehicle control was subtracted and the percentage control was calculated as the absorbance of the treated cells/control cells.

To detect the changes in intracellular ROS levels, 2′,7′‐dichlorofluorescein diacetate (DCFH‐DA, Sigma–Aldrich) assay was used. The cells (either SH‐SY5Y or C6 cells) were seeded at a density of 2.5 × 10^4^ cells per well in 48 well plates and were allowed to attach overnight. The medium was then replaced with fresh medium containing either Au@GSH (100 nM), Au@GSH‐Gal (100 nM), free GSH (500 μM), or free Gal (100 μM). After 24 h, hydrogen peroxide (2 mM) was added to the wells for 1 h to trigger an oxidative stress. After the treatment, the cells were washed once with fresh DMEM and twice with 1x PBS. Then, they were incubated with DCFH‐DA (10 μM) for 30 min and rinsed with PBS. The radioimmunoprecipitation assay (RIPA) buffer was added to each well. The collected cells were incubated at −80 °C for 20 min and then centrifuged at 21 130 g for 10 min at 4 °C. Then, the collected supernatant was transferred to a black 96‐well plate, and the fluorescence intensity was measured using the CLARIO star Plus microplate reader at an excitation wavelength of 485 nm and an emission wavelength of 530 nm. Then, 5 μL of supernatant was transferred to a transparent 96‐well plate containing 195 μL of the protein assay solution to measure the protein concentration by the BCA assay. The fluorescence intensity was normalized to the protein concentration.

For the intracellular quantification of the NPs, the cells (2 × 10^5^ cells) were seeded in each well of a 6‐well plate (in 2 mL of culture medium). After 24 h, the medium was replaced with 2 mL of a fresh medium containing either Au@GSH or Au@GSH‐Gal at a nanoparticle concentration of 25 nM. In the case of the cells incubated with Au@GSH‐Gal, a competitive test in which the cells were pretreated with 1 mM galactose was also included. Each sample was triplicated. Two incubation times, 2 and 24 h, were considered. Then, the samples were processed for elemental analysis. In detail, the cells were washed three times with PBS and trypsinized. The cell suspension was then centrifuged, the supernatant was removed, and a concentrated solution of HCl/HNO_3_ (3/1, 1 mL) was added to digest the cells. The intracellular Au concentration was measured by means of elemental analysis using a Varian 720‐ES inductively coupled plasma atomic emission spectrometer (ICP‐AES).

For optical imaging, the cells (1 × 10^5^ cells) were seeded in each well of a 12‐well plate (in 1.5 mL of culture medium). After 24 h, the medium was replaced fresh medium containing either Au@GSH or Au@GSH‐Gal at a nanoparticle concentration of 25 nM. In the case of the cells incubated with Au@GSH‐Gal a competitive test in which the cells were pretreated with 1 mM galactose was also included. After 24 h incubation, the medium was removed, and the cells were washed three times with PBS and fixed with paraformaldehyde. Finally, the cells were washed with PBS three times and imaged under an Evos M7000 optical microscope.

For the ultrastructural analysis, the cells (1 × 10^6^ cells) were seeded in each well of a 6‐well plate (in 2 mL of culture medium). After 24 h of incubation at 37 °C, the medium was replaced with a fresh medium containing Au@GSH. The cells were then incubated at 37 °C for either 1 or 24 h. Then, they were washed with PBS and fixed with glutaraldehyde (2.5%) in cacodylate buffer (0.1 M) at 4 °C for 30 min. The fixed specimens were washed three times with the same buffer, and 1% osmium tetroxide in a cacodylate buffer was added for 1 h. Next, the cells were washed and dehydrated with 25, 50, 75 and 100% acetone. Three steps of infiltration in a mixture of resin/acetone (1/2, 1/1, and 2/1 ratios) were performed, and then the specimens were embedded in 100% resin at 60 °C for 48 h. Ultrathin sections (70 nm thick) were cut on an ultramicrotome and observed under the JEOL Jem1011 electron microscope.

##### Statistical Analysis

The significance of differences among mean values was evaluated by ANOVA tests followed by post hoc Tukey's analysis for multiple comparisons. Normality of the data was examined through the Shapiro–Wilk test. Values of *p* < 0.05 were considered statistically significant.

## Conflict of Interest

The authors declare no conflict of interest.

## Supporting information

Supplementary Material

## Data Availability

The data that support the findings of this study are available from the corresponding author upon reasonable request.

## References

[1] W. Zhang , D. Xiao , Q. Mao , H. Xia , Signal. Transduct. Target Ther. 2023, 8, 267. 37433768 10.1038/s41392-023-01486-5 PMC10336149

[2] a) W. M. Pardridge , J. Cereb , Blood Flow Metab. 2012, 32, 1959; 10.1038/jcbfm.2012.126 PMC3494002 22929442

[3] X. An , W. Yu , J. Liu , D. Tang , L. Yang , X. Chen , Cell Death Dis. 2024, 15, 556. 39090114 10.1038/s41419-024-06939-5 PMC11294602

[4] M. Orlicka‐Płocka , A. Fedoruk‐Wyszomirska , D. Gurda‐Woźna , P. Pawelczak , P. Krawczyk , M. Giel‐Pietraszuk , G. Framski , T. Ostrowski , E. Wyszko , Antioxidants 2021, 10, 950. 34204594 10.3390/antiox10060950 PMC8231124

[5] a) J. B. Schulz , J. Lindenau , J. Seyfried , J. Dichgans , Eur. J. Biochem. 2000, 267, 4904; 10931172 10.1046/j.1432-1327.2000.01595.x

[6] a) N. Traverso , R. Ricciarelli , M. Nitti , B. Marengo , A. L. Furfaro , M. A. Pronzato , U. M. Marinari , C. Domenicotti , Oxid. Med. Cell. Longev. 2013, 2013, 972913; 23766865 10.1155/2013/972913 PMC3673338

[7] P. K. Mandal , D. Shukla , M. Tripathi , L. Ersland , J. Alzheimers Dis. 2019, 68, 531. 30776003 10.3233/JAD-181054

[8] a) A. Gaballo , A. Ragusa , C. Nobile , N. Gallo , L. Salvatore , C. Piccirillo , A. Nito , A. Caputo , G. Guida , A. Zito , R. Filotico , A. Quarta , Mol. Pharmaceutics 2023, 20, 5593; 10.1021/acs.molpharmaceut.3c00494 PMC10630953 37755323

[9] M. M. Rebanda , S. Bettini , L. Blasi , A. Gaballo , A. Ragusa , A. Quarta , C. Piccirillo , Nanomaterials 2022, 12, 1550. 35564258 10.3390/nano12091550 PMC9103935

[10] a) M. S. Zafar , A. Quarta , M. Marradi , A. Ragusa , Pharmaceutics 2019, 11, 505; 31581497 10.3390/pharmaceutics11100505 PMC6835330

[11] I. Cacciatore , L. Baldassarre , E. Fornasari , A. Mollica , F. Pinnen , Oxid. Med. Cell Longev. 2012, 2012, 240146. 22701755 10.1155/2012/240146 PMC3372378

[12] E. C. Dreaden , A. M. Alkilany , X. Huang , C. J. Murphy , M. A. El‐Sayed , Chem. Soc. Rev. 2012, 41, 2740. 22109657 10.1039/c1cs15237h PMC5876014

[13] C. H. Choi , C. A. Alabi , P. Webster , M. E. Davis , Proc. Natl. Acad. Sci. U.S.A. 2010, 107, 1235. 20080552 10.1073/pnas.0914140107 PMC2824286

[14] a) X. Huang , I. H. El‐Sayed , W. Qian , M. A. El‐Sayed , J. Am. Chem. Soc. 2006, 128, 2115; 16464114 10.1021/ja057254a

[15] a) N. Khlebtsov , L. Dykman , Chem. Soc. Rev. 2011, 40, 1647; 21082078 10.1039/c0cs00018c

[16] a) E. Nance , S. H. Pun , R. Saigal , D. L. Sellers , Nat. Rev. Mater. 2022, 7, 314; 38464996 10.1038/s41578-021-00394-w PMC10923597

[17] M. A. Malvindi , R. Di Corato , A. Curcio , D. Melisi , M. G. Rimoli , C. Tortiglione , A. Tino , C. George , V. Brunetti , R. Cingolani , T. Pellegrino , A. Ragusa , Nanoscale 2011, 3, 5110. 22037807 10.1039/c1nr10797f

[18] a) N. S. Aminabad , M. Farshbaf , A. Akbarzadeh , Cell Biochem. Biophys. 2019, 77, 123; 30570696 10.1007/s12013-018-0863-4

[19] M. Brust , J. Fink , D. Bethell , D. J. Schiffrin , C. Kiely , J. Chem. Soc., Chem. Commun. 1995, 1655.

[20] S. Honary , F. Zahir , Trop. J. Pharm. Res. 2013, 12, 265.

[21] L. Zhu , C. Yang , J. Qin , Chem. Commun. 2008, 6303. 10.1039/b815431g 19048136

[22] K. D. Trotter , O. Owojaiye , S. P. Meredith , P. E. Keating , M. D. Spicer , J. Reglinski , C. M. Spickett , BioMetals 2019, 32, 627. 31098734 10.1007/s10534-019-00198-0 PMC6647504

[23] E. Kaiser , R. L. Colescott , C. D. Bossinger , P. I. Cook , Anal. Biochem. 1970, 34, 595. 5443684 10.1016/0003-2697(70)90146-6

[24] K. Klein , K. Loza , M. Heggen , M. Epple , ChemNanoMat. 2021, 7, 1330.

[25] M. Le Goas , J. Saber , S. G. Bolívar , J.‐M. Rabanel , J.‐M. Awogni , D. C. Boffito , X. Banquy , Nano Today 2022, 45, 101516.

[26] a) S. Nagamatsu , Y. Nakamichi , N. Inoue , M. Inoue , H. Nishino , H. Sawa , Biochem. J. 1996, 319, 477; 8912684 10.1042/bj3190477 PMC1217793

[27] X. Guan , Med. Chem. Res. 2024, 33, 1281.

[28] M. Liu , M. Sharma , G. Lu , Z. Zhang , W. Song , J. Wen , Pharmaceutics 2025, 17, 4. 10.3390/pharmaceutics17010004 PMC11768106 39861655

[29] H. Nosrati , M. Tarantash , S. Bochani , J. Charmi , Z. Bagheri , M. Fridoni , M.‐A. Abdollahifar , S. Davaran , H. Danafar , H. Kheiri Manjili , ACS Biomater. Sci. Eng. 2019, 5, 1677.

[30] a) A. Grover , A. Hirani , Y. Pathak , V. Sutariya , AAPS PharmSciTech 2014, 15, 1562; 25134466 10.1208/s12249-014-0165-0 PMC4245440

[31] a) J. N. Reginald‐Opara , M. Tang , D. Svirskis , L. Chamley , Z. Wu , Int. J. Pharm. 2022, 626, 122152; 36055442 10.1016/j.ijpharm.2022.122152

[32] a) C. Zhou , G. Hao , P. Thomas , J. Liu , M. Yu , S. Sun , O. K. Öz , X. Sun , J. Zheng , Angew. Chem. Int. Ed. Engl. 2012, 51, 10118; 22961978 10.1002/anie.201203031

[33] a) D. Luo , X. Wang , C. Burda , J. P. Basilion , Cancers 2021, 13, 1825; 33920453 10.3390/cancers13081825 PMC8069007

[34] B. Pradhan , S. Patra , C. Behera , R. Nayak , B. P. Jit , A. Ragusa , M. Jena , Molecules 2021, 26, 1171. 33671811 10.3390/molecules26041171 PMC7926928

