# Supplementary material for: Galactose‐Functionalized Gold Nanoparticles Targeting Membrane Transporters for the Glutathione Delivery to Brain Cancer Cells
Source: Chembiochem. 2025 Jun 17;26(13):e202500104. doi: 10.1002/cbic.202500104 (PMC12247028; doi:10.1002/cbic.202500104)
Supplement: Supplementary file 1 — Supplementary Material [file CBIC-26-e202500104-s001.pdf]

Galactose-Functionalized Gold Nanoparticles Targeting Membrane Transporter for the Glutathione Delivery to Brain Cancer Cells

Francesca Milano,[a] Alessia Nito,[b,c] Annalisa Caputo,[b,d] Antonio Gaballo,[b] Marco Marradi,[a] Alessandra Quarta*[b] and Andrea Ragusa*[b,e]

[a] F. Milano, Prof. M. Marradi
Department of Chemistry 'Ugo Schiff'
University of Florence
via della Lastruccia 3-13
50019 Sesto Fiorentino (FI), Italy
E-mail: francesca.milano@unifi.it
E-mail: marco.marradi@unifi.it

[b] A. Nito, A. Caputo, Dr. A. Gaballo, Dr. A. Quarta
CNR NANOTEC, Institute of Nanotechnology
Campus Ecotekne
73100, Lecce, Italy
E-mail: antonio.gaballo@nanotec.cnr.it
E-mail: alessandra.quarta@nanotec.cnr.it

[c] A. Nito
Department of Engineering for Innovation
University of Salento
Campus Ecotekne
73100, Lecce, Italy
E-mail: alessia.nito@unisalento.it

[d] A. Caputo
Department of Mathematics and Physics
University of Salento
Campus Ecotekne
73100, Lecce, Italy
E-mail: annalisa.caputo@unisalento.it

[e] Prof. A. Ragusa
Department of Life Sciences, Health and Health Professions
Link Campus University
Via del Casale di San Pio V, 44
00165 Rome, Italy
E-mail: a.ragusa@unilink.it

Supporting Information

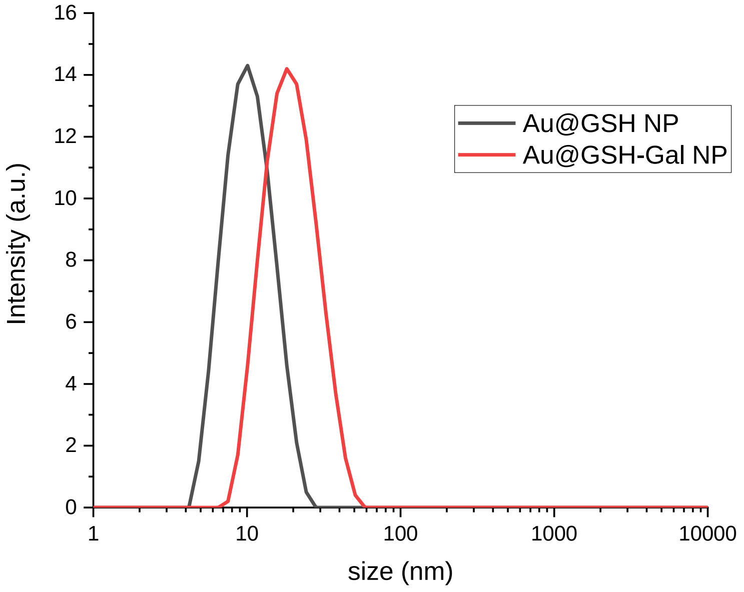

Figure S1. Size distribution of the Au@GSH and Au@GSH-Gal NPs in H_2O at 25 °C as determined by DLS.

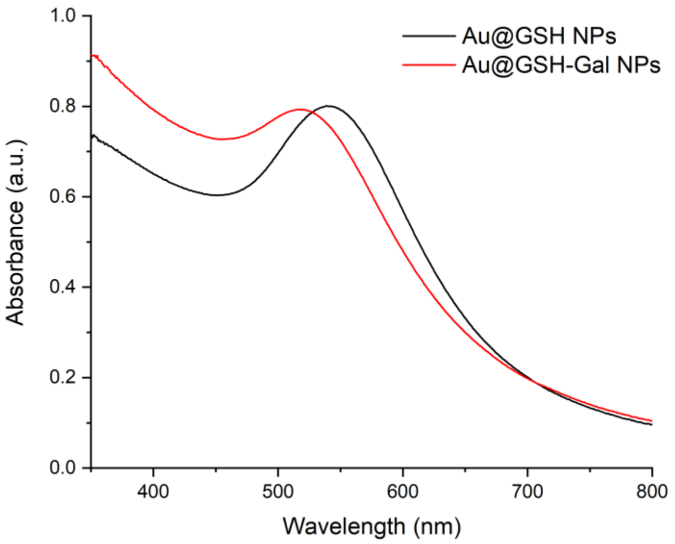

Figure S2. Absorbance spectra of the Au@GSH and Au@GSH-Gal NPs as determined by UV-vis.

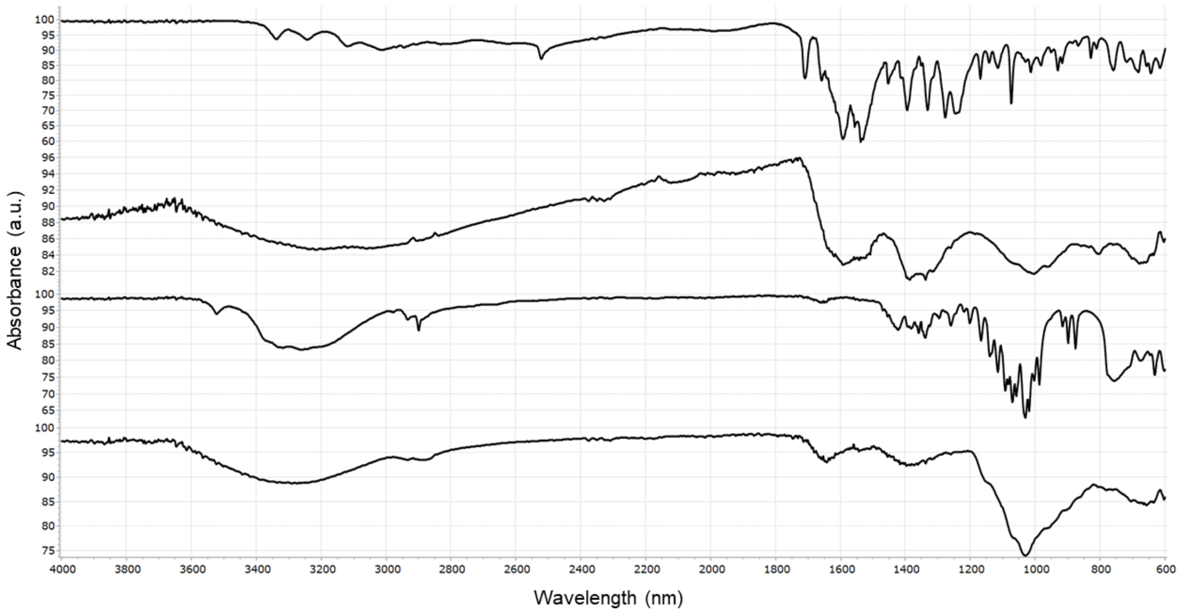

Figure S3. FT-IR spectra of the (from top to bottom) GSH, Au@GSH NPs, lactose, and Au@GSH-Gal NPs.

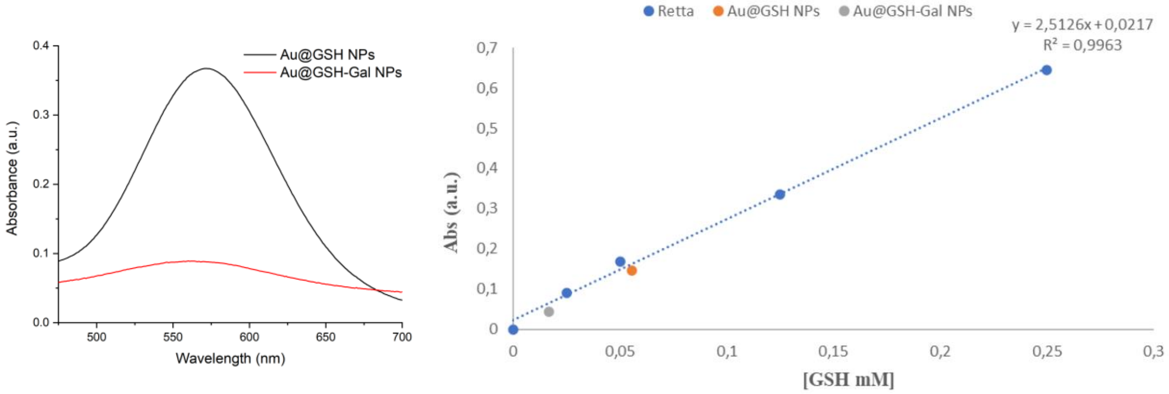

Figure S4. a) Absorbance spectra obtained by the ninhydrin test before (Au@GSH NPs, black line) and after (Au@GSH-Gal NPs, red line) functionalization with galactose. b) Calibration curve with GSH (blue dots) for the Kaiser test and corresponding values obtained for the Au@GSH (orange dots) and the Au@GSH-Gal NPs (grey dots).

Table S1. Estimation of the S/Au and S/Au NP molar ratio as determined by ICP elemental analysis.

S/Au molar ratio	S/Au NP molar ratio
0.62 ± 0.10	3335 ± 519

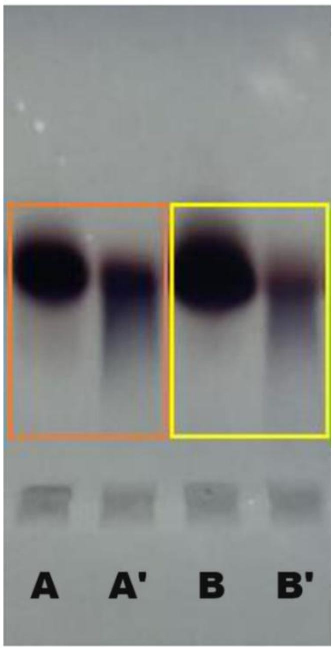

Figure S5. Agarose gel electrophoresis image representing the Au@GSH (A and B) and Au@GSH-Gal NPs (A’ and B’) from two different preparations (orange and yellow boxes, respectively).
